# Care Coordination for Patients Admitted With Alcohol-Associated Liver Disease: An Assessment of Appropriate Follow-up and Treatment at Time of Discharge

**DOI:** 10.1016/j.gastha.2025.100823

**Published:** 2025-09-30

**Authors:** Isabelle S. Byers, Bharathi Selvan, Jacqueline B. Henson, Donna Niedzwiecki, Andrew J. Muir, Stephanie Garbarino

**Affiliations:** 1 Department of Medicine, Duke University School of Medicine, Durham, North Carolina; 2 Division of Gastroenterology, Department of Medicine, Duke University School of Medicine, Durham, North Carolina; 3 Department of Biostatistics and Bioinformatics, Duke University School of Medicine, Durham, North Carolina

**Keywords:** alcohol-associated liver disease, alcohol use disorder, care coordination

## Abstract

**Background and Aims:**

The prevalence of alcohol-associated liver disease (ALD) is on the rise in the United States, and effective management requires multidisciplinary care and coordination of services across specialties. The aim of this study is to assess whether patients hospitalized with alcohol use disorder (AUD) and ALD have access to appropriate provider follow-up and treatment at time of discharge as well as to identify factors that enable care coordination.

**Methods:**

Hospital admissions for adults with ALD and AUD between January 1, 2022, and December 31, 2022, at our institution were identified. Medical records were reviewed to identify clinical characteristics as well as attendance at a hepatology appointment within 90 days of discharge (primary endpoint), referral to substance use services at discharge, and prescription of medication for AUD at discharge (secondary endpoints). Logistic regression modeling with repeated admissions within patient was performed to identify independent associations with each outcome.

**Results:**

Among the hospital admissions, 417 met the inclusion criteria. Following 16% of admissions, patients attended a hepatology appointment, 4% of patients were referred to substance use services, and 14% of patients were prescribed medication for AUD. Among modifiable factors, hepatology and social work evaluation during hospitalization were associated with successful care coordination.

**Conclusion:**

In our population, rates of specialist follow-up and treatment for AUD and ALD after a hospital admission were overall low. This study not only highlights a significant gap in care delivery for patients with ALD, but it also identifies a critical need to establish quality measures for the inpatient management and long-term follow-up for patients ALD.

## Introduction

Alcohol use disorder (AUD) and alcohol-associated liver disease (ALD) are complex chronic conditions with significant morbidity. ALD is associated with long-term alcohol use^1^ and includes a spectrum of alcohol-induced liver pathology ranging from steatosis and steatohepatitis to progressive fibrosis. Unfortunately, most patients with ALD are diagnosed at advanced stages following the development of alcohol-related hepatitis (AH), alcohol-related cirrhosis, or hepatocellular carcinoma, when symptoms of decompensated liver disease or liver failure manifest and treatment options are limited.^2^^,^^3^ Delays in diagnosis of ALD and treatment of the underlying substance use disorder (SUD) have led to poor outcomes in this patient population such that AUD is responsible for half of all cirrhosis-related deaths, and ALD is the most common indication for liver transplantation in the United States.^4^, ^5^, ^6^

Improving outcomes for patients with ALD relies on multidisciplinary management of both the patient’s liver disease and comorbid AUD and requires early involvement of specialists in hepatology, addiction/psychiatry, and social work.^7^^,^^8^ In a systematic review of treatment trials for ALD, integrating AUD treatment providers alongside medical providers in clinic produced better abstinence rates than usual care.^7^ Another study found that patients hospitalized with AUD without a known history of liver disease were more likely to undergo liver fibrosis screening and advanced fibrosis identification, receive AUD pharmacotherapy prescription, and receive preventative hepatology care when evaluated by both hepatology and addiction consultation teams compared to addiction consultation alone.^9^ While the American Association for the Study of Liver Diseases practice guidelines highlight the importance of multidisciplinary care for patients with ALD and AUD,^8^ little is known about the implementation of these practice standards at individual healthcare institutions.

The objective of this study is to assess multidisciplinary care coordination for individuals hospitalized with ALD and AUD at our institution. Hospitalization represents a pivotal time for providers to intervene earlier in the disease course, coordinate multidisciplinary care and provider follow-up, and initiate evidence-based treatment. Our primary aim was to determine whether patients hospitalized with ALD and AUD attend a hepatology appointment within 90 days of discharge. Our secondary aims were to determine whether patients hospitalized with ALD and AUD are referred to substance use services and prescribed medications for alcohol use disorder (MAUD) at time of discharge. Further, we sought to identify sociodemographic and clinical characteristics associated with each of these outcomes.

## Materials and Methods

### Design and Data Collection

**Figure 1:**
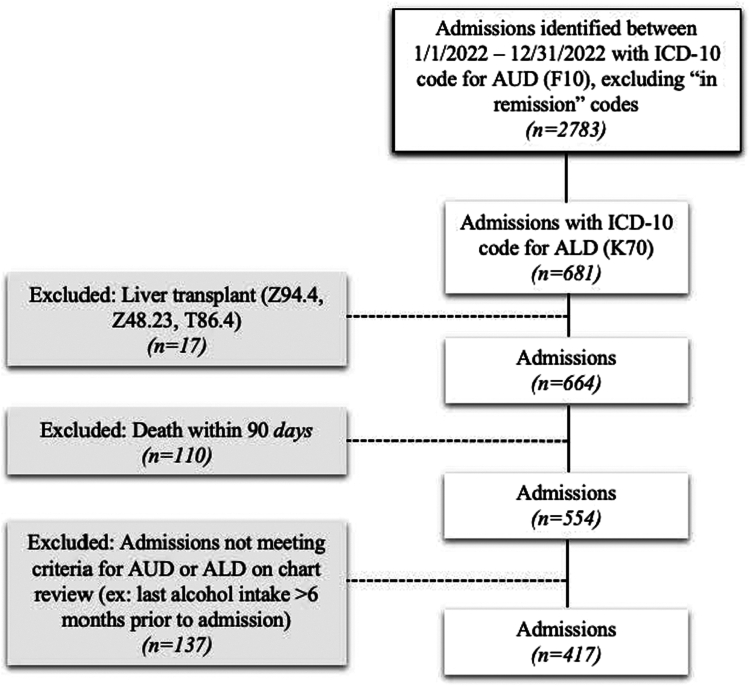
Flowchart for study inclusion criteria.

We conducted a single-center retrospective cohort study that included all patients admitted to the Duke University Health System between January 1, 2022, and December 31, 2022, with both AUD and ALD. The Duke University Health System includes 1 tertiary referral center and 2 community-based hospitals. AUD was defined using International Classification of Diseases (ICD)-10 code F10, and ALD was defined using ICD-10 codes K70.0-K70.9, which include both alcohol-related hepatitis and alcohol-related cirrhosis. Individuals with ICD-10 codes for AUD “in remission” (F10.11, F10.21, and F10.91), those with a history of liver transplantation (Z94.4, Z48.23, T86.4), and those who were deceased within 90 days of discharge were excluded from the study. Manual chart review was conducted to verify that individuals met the diagnostic criteria for AUD and ALD. Individuals without recent alcohol use within 6 months prior to admission, either based on self-report or alcohol biomarkers (PEth or serum toxicity screen), were also excluded. If individuals denied recent alcohol use on self-report but alcohol biomarkers were found to be positive, these individuals were included in the study population (Figure 1).

Patient demographics (age, sex, race, ethnicity, insurance, address) and encounter information (admission and discharge dates, admitting service) were extracted from the electronic health record. Model for End Stage Liver Disease (MELD) -Na score was calculated based on laboratory values on admission. Substance use history including recent alcohol use, prior history of complicated alcohol withdrawal (defined as either seizures or delirium tremens), and prior history of pharmacologic treatment for AUD were obtained through manual chart review. Brand and generic names for naltrexone, acamprosate, disulfiram, gabapentin, topiramate, and baclofen were inputted into our electronic health record’s search feature to identify prior pharmacologic treatment for AUD. Manual chart review was also performed to evaluate whether patients had previously been seen by a hepatologist in the outpatient setting. Due to the fragmented nature of behavioral health care and its lack of integration into our electronic medical record, we were unable to systematically collect information regarding patients’ behavioral health history (ie whether a patient had previously accessed substance use services or recovery-based treatment).

Manual chart review was performed to assess whether hepatology was consulted during admission. Our institution does not house a dedicated inpatient hepatology service, and patients with liver disease are typically admitted to general medicine with hepatology or gastroenterology teams serving as consultants. As 2 of the 3 clinical sites in this study do not have a dedicated hepatology consult service, general gastroenterology consults were considered as hepatology consults if ALD was specifically addressed by the consultant. At all clinical sites, hepatology/gastroenterology consults services were available 7 days a week.

Manual chart review was also performed to assess whether patients received dedicated substance use counseling during admission. While our institution does not house dedicated addiction medicine or addiction psychiatry services, a clinical social work team exists to provide counseling and resources to patients with SUD. Specifically, this team is responsible for screening individuals for SUD, providing brief motivational interviewing and behavioral counseling, and coordinating outpatient resources and referrals for patients who are interested. Of note, this team does not prescribe pharmacologic treatment for AUD. Not all patients who are hospitalized with active SUD are evaluated by our clinical social work team. Rather, this service is consult-based and involved at the discretion of the primary team. While a consult order may be placed in the electronic medical record any time, the clinical social work team evaluates patients between 8 am–5 pm Mondays to Fridays.

Outcome measures collected included attendance at a hepatology appointment within 90 days of discharge, referral to substance use services at discharge, and prescription of MAUD at discharge. Attendance at an outpatient hepatology appointment was selected as an outcome measure rather than whether an appointment was scheduled to more accurately assess care coordination and patient access. Further, not every patient who follows up with hepatology has had an appointment scheduled at time of hospital discharge. Manual chart review was performed to determine whether patients attended a hepatology appointment within 90 days of discharge.

Manual chart review was also performed to evaluate whether patients hospitalized with AUD and ALD were referred to substance use services at time of discharge. Referral to substance use services was selected as an outcome measure rather than patient utilization of these resources as utilization trends of community-based resources are not ascertainable through the electronic medical record. Referrals to substance use services were identified in 1 of 3 ways. First, for patients evaluated by the inpatient clinical social work team, consult notes were directly reviewed. Second, ambulatory referrals to “adult behavioral health” were tracked. Although this is not a standardized workflow process, primary teams occasionally place this referral at time of discharge for patients with SUD. Third, the patient instructions section of discharge paperwork was reviewed to identify what SUD services patients were offerred. In each of these cases, depending on severity of AUD and insurance status, patients can be referred to a range of services including outpatient substance use counseling (1-on-1 psychotherapy, motivational enhancement therapy, cognitive behavior therapy, or group therapy), community mutual aid societies (such as Alcoholics Anonymous), intensive outpatient treatment or residential substance use treatment.

Discharge summaries were reviewed to identify whether MAUD was newly prescribed during admission or continued from prior. MAUD included any 1 of the 3 medications approved by the Food and Drug Administration for AUD (naltrexone, acamprosate, or disulfiram) as well as medications used off-label for AUD (gabapentin, topiramate, and baclofen).

### Statistical Analysis

Descriptive statistics were computed for demographic and clinical characteristics. Continuous variables were reported as mean and standard deviation or median and interquartile range. Categorical variables were reported as frequency and corresponding percentage. The proportion of patients meeting outcome measures was compared by demographic and clinical characteristics. Logistic regression modeling with repeated admissions within patient was performed to identify associations with demographic and clinical characteristics in univariate and multivariable analyses.

## Results

### Characteristics of Clinical Encounters

During the study period, there were a total of 417 admissions for AUD and ALD in 318 patients. The mean age of patients was 50 years (Table 1). The majority of admissions involved patients who identified as male (71%), non-Hispanic (89%), and White (57%). Most patients also had health insurance of some kind (77.8%). The General Medicine service was the primary admitting service in nearly all cases (94%). Patients required intensive care in 10% of admissions, most frequently due to the severity of their liver disease. The mean MELD-Na was 18. In 23% of admissions, patients had a prior history of complicated alcohol withdrawal. Some patients had previously established care with hepatology in the outpatient setting (31%) or had previously been prescribed MAUD (30%). In 35% of admissions, patients were evaluated by hepatology for management of ALD. In 38% of admissions, patients were evaluated by the clinical social work team for substance use counseling (Table 1).

**Table 1:** Demographic and Clinical Characteristics of Admissions for AUD and ALD

Characteristic	N = 417
Age, mean (SD), y	50.1 (11.4)
Sex, N (%)	
Female	120 (28.64)
Male	299 (71.36)
Ethnicity, N (%)	
Hispanic	47 (11.41)
Non-Hispanic	365 (88.59)
Race, N (%)	
Black	138 (34.24)
Other	35 (8.68)
White	230 (57.07)
Insurance, N (%)	
Commercial	152 (36.28)
Medicaid	126 (30.07)
Medicare	33 (7.88)
Other	15 (3.58)
Self-Pay	93 (22.20)
Admitting service, N (%)	
Medicine	395 (94.27)
Other	24 (5.73)
Admission source, N (%)	
Nontransfer	373 (89.02)
Outside hospital transfer	46 (10.98)
ICU admission, N (%)	45 (10.74)
Reason for ICU admission	
Liver disease	18 (40.00)
Alcohol use disorder	11 (24.4)
Other	16 (35.56)
MELD-Na, mean (SD)	18.49 (7.61)
Prior complicated withdrawal, N (%)	94 (22.65)
Prior hepatology visit, N (%)	129 (30.79)
Prior pharmacologic treatment for AUD, N (%)	125 (29.83)
Hepatology evaluation during hospitalization, N (%)	148 (35.32)
Clinical social work evaluation during hospitalization, N (%)	159 (37.95)

### Multidisciplinary care coordination

After 68 (16%) admissions, patients attended a hepatology follow-up within 90 days of discharge. Factors associated with hepatology appointment attendance included a prior hepatology appointment (*P* = .0002), hepatology evaluation for ALD during hospitalization (*P* < .0001), and higher MELD-Na score (*P* = .009) (Table 2). In multivariable analysis, prior hepatology appointment (*P* = .02) and hepatology evaluation for ALD during hospitalization (*P* = .02) remained significant while higher MELD-Na score (*P* = .32) did not.

**Table 2:** Outcomes

Characteristic	Hepatology follow-up within 90 d			Referral to substance use services			Rx for MAUD at discharge		
	Yes (N = 68)	No (N = 349)	*P* value^a^	Yes (N = 17)	No (N = 400)	*P* value^a^	Yes (N = 57)	No (N = 360)	*P* value^a^
Age, mean (SD), y	48.4 (10.8)	50.5 (11.4)	.21	46.1 (9.9)	50.3 (11.4)	.13	48.5 (10.6)	50.4 (11.5)	.16
Sex, N (%)			.92			.24			.69
Female	19 (16%)	100 (84%)		7 (5.9%)	112 (94.1%)		18 (15.1%)	101 (84.9%)	
Male	49 (16.4%)	249 (83.6%)		10 (3.4%)	288 (96.6%)		39 (13.1%)	259 (86.9%)	
Ethnicity, N (%)			.70			.11			.23
Hispanic	9 (19.1%)	38 (80.9%)		4 (8.5%)	43 (91.5%)		9 (19.1%)	38 (80.9%)	
Non-Hispanic	58 (16%)	305 (84%)		13 (3.6%)	350 (96.4%)		47 (12.9%)	316 (87.1%)	
Race, N (%)									
Black vs white			.28			.97			.40
Other vs white			.72			.74			.85
Black	17 (12.6%)	118 (87.4%)		6 (4.4%)	129 (95.6%)		22 (16.3%)	113 (83.7%)	
Other	8 (22.9%)	27 (77.1%)		1 (2.6%)	37 (97.4%)		5 (13.2%)	33 (86.8%)	
White	40 (17.5%)	188 (82.5%)		9 (3.9%)	219 (96.1%)		28 (12.3%)	200 (87.7%)	
Admitting service, N (%)			.57			.98			**.04∗**
Medicine	63 (16%)	330 (84%)		16 (4.1%)	377 (95.9%)		57 (14.5%)	336 (85.5%)	
Other	5 (20.8%)	19 (79.2%)		1 (4.2%)	23 (95.8%)		0 (0%)	24 (100%)	
ICU admission, N (%)			.26			.51			.06
Yes	10 (22.2%)	35 (77.8%)		1 (2.2%)	44 (97.8%)		2 (4.4%)	43 (95.6%)	
No	58 (15.6%)	314 (84.4%)		16 (4.3%)	356 (95.7%)		55 (14.8%)	317 (85.2%)	
MELD-Na, mean (SD)	21.0 (8.5)	17.9 (7.3)	**.009**	17.7 (8.1)	18.5 (7.6)	.52	19.0 (7.6)	15.0 (6.6)	**.0004**
Prior complicated withdrawal, N (%)			.09			**.01**			.12
Yes	10 (10.6%)	84 (89.4%)		8 (8.5%)	86 (91.5%)		17 (18.1%)	77 (81.9%)	
No	58 (18.1%)	263 (81.9%)		9 (2.8%)	312 (97.2%)		38 (11.8%)	283 (88.2%)	
Prior hepatology visit, N (%)			**.0002**			.89			.61
Yes	37 (28.7%)	92 (71.3%)		5 (3.9%)	124 (96.1%)		16 (12.4%)	113 (87.6%)	
No	31 (10.8%)	257 (89.2%)		12 (4.2%)	276 (95.8%)		41 (14.2%)	247 (85.8%)	
Prior pharmacologic treatment for AUD, N (%)			.52			.11			**<.0001**
Yes	18 (14.5%)	106 (85.5%)		8 (6.4%)	116 (93.6%)		35 (28.2%)	89 (71.8%)	
No	50 (17.1%)	243 (82.9%)		9 (3.1%)	284 (96.9%)		22 (7.5%)	271 (92.5%)	
Hepatology evaluation during hospitalization, N (%)			**<.0001**			.29			.06
Yes	45 (30.4%)	103 (69.6%)		4 (2.7%)	144 (97.3%)		14 (9.5%)	134 (90.5%)	
No	23 (8.6%)	246 (91.4%)		13 (4.8%)	256 (95.2%)		43 (16%)	226 (84%)	
Clinical social work evaluation during hospitalization, N (%)			.91			**.01**			**.0007**
Yes	26 (16.6%)	131 (83.4%)		11 (7%)	146 (93%)		33 (21%)	124 (79%)	
No	42 (16.2%)	218 (83.8%)		6 (2.3%)	254 (97.7%)		24 (9.2%)	236 (90.8%)	

Bold entries indicate significance.

a Except where indicated by an asterisk (∗), *P* values are based on a generalized regression model using a logit linking function with repeated measures within patient.

Patients were referred to substance use services at the time of discharge in 17 (4%) admissions. Factors associated with a referral to substance use services included history of complicated alcohol withdrawal (*P* = .01) and clinical social work evaluation for substance use counseling during hospitalization (*P* = .05) (Table 2). In multivariable analysis, history of complicated alcohol withdrawal (*P* = .05) remained significant while clinical social work evaluation for substance use counseling during hospitalization (*P* = .06) did not.

Patients were prescribed MAUD at hospital discharge in 57 (14%) admissions. Most patients were prescribed naltrexone (67%), gabapentin (18%), or acamprosate (15%). Factors associated with AUD prescription at discharge included prior pharmacologic treatment for AUD (*P* < .0001), admission to the general medicine service (*P* = .04), higher MELD-Na score (*P* = .0004), and clinical social work evaluation for substance use counseling during hospitalization (*P* = .0007) (Table 2). In multivariable analysis, prior pharmacologic treatment for AUD (*P* < .0001) and higher MELD-Na score (*P* = .006) remained significant, while clinical social work evaluation for substance use counseling during hospitalization (*P* = .06) did not. Admission to the general medicine service was omitted from this analysis due to computational errors in fitting the statistical model. Demographic characteristics, including age, race, ethnicity, insurance status and distance from the hospital, were not significantly associated with primary or secondary outcomes.

## Discussion

### Hepatology Follow-up

Multidisciplinary care for AUD and ALD is recommended, though the extent to which this occurs in practice is uncertain. In this study, we examined the standard of care for patients hospitalized with ALD and AUD in our health system. We found that among individuals hospitalized with ALD and AUD, rates of hepatology follow-up were overall low as roughly 1 in 6 individuals attended an appointment within 90 days of discharge. Patients who had previously established care with hepatology in the outpatient setting or those who were evaluated by hepatology for ALD during admission were more likely to attend a follow-up. This suggests, perhaps not surprisingly, that previous interface with a specialty team facilitates coordination and continuation of specialty care in the outpatient setting. Further, inpatient hepatology evaluation may increase the identification of patients who warrant outpatient management of chronic liver disease such as individuals at high risk of decompensation or progression of fibrosis.

At our institution, most patients admitted with ALD are managed by a general medicine or ICU team, and decisions to consult the inpatient hepatology service or refer to outpatient hepatology are made on a case-by-case basis, though typically occur for more severe manifestations of liver disease. While our study suggests that care coordination in the outpatient setting is facilitated by inpatient hepatology consultation, we hesitate to recommend the involvement of hepatology for the management of *all* patients admitted with AUD and ALD, as the high incidence of these diseases could outstrip available hepatology specialty resources. Further, not all patients admitted with ALD require inpatient hepatology consultation, as is the case for patients with compensated cirrhosis or mild-moderate AH. However, standardized workflows should be established to aid primary teams in identifying which patients warrant specialist follow-up. For instance, patients not only with severe or decompensated disease, but also those with cirrhosis or advanced fibrosis should be referred to outpatient hepatology for disease surveillance and screening. Additionally, timely transitions of care between inpatient and outpatient settings should be prioritized such that prior to discharge, available outpatient specialists are identified, referrals are placed, and appointment scheduling is facilitated.

Beyond surveillance of chronic liver disease, outpatient hepatology visits offer additional opportunities to engage in management of AUD, including initiation of medications for AUD and referral to additional recovery supports. Hepatologists can proactively address these issues, ensuring that patients are managed safely and effectively over the long-term.^10^ In 1 study by Blaney et al., patients actively enrolled in inpatient alcohol treatment were more motivated to engage in treatment for relapse prevention when hepatologists were involved in their care.^11^

### Referral to Substance Use Services

Previous work has established that timely access to behavioral care for AUD following hospitalization for ALD is associated with decreased 30-day readmission, decreased 30-day alcohol relapse, and decreased mortality.^12^ Our study found that among individuals hospitalized with ALD and AUD, referral rates to substance use services at discharge were overall low at less than 5%. This is consistent with low rates identified in prior literature.^13^ At present, no standardized pathway exists for inpatient providers to refer patients to outpatient substance use resources. While some providers may choose to involve the clinical social work team to help with coordination of outpatient resources, others may choose to informally counsel patients on AUD or attach a list of community resources to patient discharge instructions. However, because community-based substance use services vary in intensity, across counties, and by insurance type, it is difficult for inpatient providers to maintain full awareness of available services.

We found that patients were more likely to be referred to substance use services at discharge if they had a history of complicated alcohol withdrawal. As these patients are at higher risk for relapse and medical complication, provider teams may be more motivated to connect patients with resources prior to discharge. However, more work needs to be done at the level of the health system to refine referral practices, such that all patients who are admitted with AUD are provided with substance use services and recovery-based treatment options at the time of discharge, not just those who have already experienced complications.

Although inpatient evaluation by the clinical social work team was not found to be significantly associated with referral to substance use services in multivariable analysis, an association was nevertheless observed. Standardizining the involvement of inpatient provider teams—such as clinical social work, addiction medicine, or addiction psychiatry—may improve rates of dedicated substance use counseling as well as referral to community resources at discharge. In practice settings where these provider teams do not exist or are overburdened, primary teams may be required to take on this role. In this case, health systems must be responsible for prioritizing provider education on effective and available substance use services.

### MAUD

Prescription of MAUD was overall low in our study, similar to trends reported in previous literature.^13^ Naltrexone was the most commonly prescribed medication, followed by gabapentin and acamprosate. Factors associated with MAUD prescription at discharge included prior pharmacologic treatment for AUD and higher MELD-Na score. Reluctance among patients and providers to begin MAUD—especially in the setting of liver disease—is well-documented.^13^ Therefore, it is not surprising that patients previously taking MAUD were more likely to have these prescriptions continued or restarted at discharge compared to individuals not previously on MAUD. This is likely a result of provider familiarity with medications for alcohol use disorder, as behavioral health providers are the primary prescribers of these medications across treatment settings. Ultimately, more efforts should be undertaken to educate medical providers on the medications available for treatment of AUD, and safe for use in patients with liver disease.

We also found that a higher MELD-Na score was associated with prescription of MAUD at discharge, which differs from prior research identifying an association between lower MELD scores and prescription of MAUD.^13^ However, in the previous study, the patient population was limited to individuals with cirrhosis, whereas our study also included individuals with alcohol-related hepatitis (AH). MELD scores may be transiently higher for these patients on admission and decrease by the time of discharge. Regardless, MAUD should not be reserved for the sickest patients with the highest MELD scores, but instead, offered to patients at the earliest possible time in the disease course.

Although inpatient evaluation by the clinical social work team was not found to be significant in multivariable analysis, an association between inpatient evaluation and prescription of MAUD at discharge indeed exists. While the clinical social work team at our institution does not directly prescribe MAUD, they provide an additional source of support to the patient, and their therapeutic alliance may increase the likelihood that a patient expresses readiness for change or is amenable to pharmacotherapy at time of discharge. As such, it would be beneficial for institutions to develop inpatient standards of care for the management of ALD whereby all patients admitted with either alcohol-associated cirrhosis or hepatitis are evaluated by a substance use counseling or addiction medicine team. Further, it would be prudent for these teams to include providers with prescribing capabilities to increase the frequency MAUD is prescribed.

## Future Implications

Ultimately, this study illustrates that patients hospitalized with ALD and AUD at our institution have low rates of hepatology follow-up, infrequent referrals to substance use services, and low utilization of of MAUD. Rather than reserving multidisciplinary resources and treatment strategies for individuals with severe manifestations of ALD, health systems ought to prioritize early care coordination for this high risk population at a pivotal time in the disease course. Actionable steps to achieve this include developing a set of quality metrics for the inpatient management of ALD, standardizing the involvement of substance use counseling and addiction medicine services, streamlining referral pathways, and educating medical providers on safe therapeutic options for the treatment of AUD in patients with liver disease.

## Limitations

As a single-center retrospective observational cohort study, limitations of this analysis include the inability to make causal inferences, the potential for unmeasured confounding variables, and limited generalizability. Although this is a single-center study, we included 3 different hospitals including a tertiary referral center and 2 more community-based hospitals which should improve generalizability. Another limitation of the study arises from the use of ICD-10 codes, which are often imprecise in diagnosis identification. We attempted to address this by utilizing individual chart reviewers to adjudicate each case. We also recognize limitations in our outcome measures. Notably, because there was no standardized method in which patients were referred to substance use services, we may have underestimated the rate of these referrals based on the imperfect nature of chart review. Additionally, it is possible that patients declined referrals to substance use services and that this preference may not have been documented in the medical record.

## Conclusion

As the prevalence of AUD and ALD continue to rise in the United States,^14^ it is imperative that healthcare systems facilitate the early coordination of multidisciplinary care and substance use disorder treatment. At our institution, patients hospitalized with ALD and AUD were infrequently connected to hepatology and substance use services and seldom prescribed MAUD at discharge. This study not only highlights a significant gap in care delivery for patients with ALD, but it also identifies a critical need to establish quality measures for the inpatient management of and long-term follow-up for patients with ALD.
